# LLM-Reflex-GeoKG: A reflexion-enhanced LLM framework for automated geographic knowledge graph construction

**DOI:** 10.1371/journal.pone.0344565

**Published:** 2026-04-02

**Authors:** Zongjun Wei, Gang Chen, Youheng Xu, Xiangfei Yang, Mengting Cao, Minyi Liao

**Affiliations:** 1 School of Geography and Ocean Science, Nanjing University, China; 2 Jiangsu Provincial Key Laboratory of Geographic Information Science and Technology, Nanjing, China; 3 Nanjing Memory Graph Cutural Technology co. Ltd, China; Minnan Normal University, CHINA

## Abstract

The automated construction of high-quality, domain-specific Geographic Knowledge Graphs (GeoKGs) is pivotal for intelligent applications, such as river network modelling, digital watershed management, and cultural heritage conservation in river basins. However, this task faces a core challenge of inaccurate and incomplete knowledge extraction. While Large Language Models (LLMs) offer a new paradigm, their direct application is often undermined by inherent hallucinations and a limited grasp of complex geospatial semantics. To address these challenges, this study proposes and validates a novel framework, LLM-Reflex-GeoKG, which integrates a Reflexion-style self-reflection loop with a collaborative dual-LLM generator–critic scheme and a multi-stage extraction strategy for GeoKG construction.Experiments on data from the Yangtze River Delta river network demonstrate that our framework achieves F1 scores of 0.898 and 0.823 on geographic entity recognition and relation extraction tasks, significantly outperforming baseline systems. This research confirms that our framework improves both the accuracy and completeness of automated knowledge acquisition while reducing reliance on extensive manual annotation, offering a practical paradigm for building reliable, domain-specific GeoKGs.

## Introduction

A Knowledge Graph (KG), which represents real-world knowledge structurally through entities and their relationships, has become a cornerstone of modern artificial intelligence [1]. KGs are generally categorized into two types: general-purpose KGs that cover extensive commonsense knowledge [2], and domain-specific KGs tailored to particular industries [3]. While the former focuses on broad encyclopedic knowledge, the latter contends with more complex knowledge structures and greater construction challenges due to the heterogeneity of specialized data [4]. In recent years, the Geographic Knowledge Graph (GeoKG) has emerged as a vital class of domain-specific KG. It has demonstrated significant value in integrating and analyzing complex geospatial data, thereby supporting critical applications in smart cities, resource management, and—as the focus of this study—water network management [5–8]. These domain-specific tasks fall within the spectrum of spatially explicit decision support systems and demand rigorous spatial precision and specialized semantic modeling, far exceeding the requirements of general-purpose information retrieval. To effectively handle the professional terminology and complex relationships inherent to such regions, it is of paramount importance to develop dedicated methods for efficiently and accurately constructing high-quality GeoKGs from massive unstructured geographic texts, thereby enabling intelligent querying, in-depth analysis, and seamless integration with downstream applications [9–11].

The construction of a reliable GeoKG hinges on effective Knowledge Extraction (KE) [12]. This process involves two key sub-tasks: Named Entity Recognition (NER), for locating geographic entities within text [13], and Relation Extraction (RE), for identifying the semantic and spatial connections between these entities [14]. Prior to the LLM era, traditional GeoKG construction methods, which rely on expert-curated knowledge bases [15] or supervised NLP techniques (e.g., BERT-CRF) [16], established the state-of-the-art in precision for closed-domain tasks. However, these methods are often constrained by their heavy reliance on large-scale annotated datasets and limited adaptability to the complexity and dynamic nature of geographic information. These limitations are particularly acute in the laborious and costly processes of data source interpretation, entity-relation definition, rule formulation, data annotation, and knowledge validation, hindering their scalability for large-scale, dynamically updated tasks such as water network knowledge graph construction. [17–19].Recently, the advent of Large Language Models (LLMs) like GPT has led to breakthroughs in natural language understanding, semantic reasoning, and text generation [20,21], presenting new opportunities for automated KG construction [22]. The formidable context-modeling and language-generation capabilities of LLMs show immense potential for processing unstructured text and reducing the dependence on explicit annotations [23], with promising initial results in general-purpose KG construction tasks [24].

In the context of Named Entity Recognition (NER), LLMs have been widely applied to diverse domain-specific corpora for identifying entities such as proper nouns, locations, and organizations, achieving commendable performance in zero-shot or few-shot settings. Existing research has leveraged mechanisms like task prompting, instruction fine-tuning, and contrastive learning to enhance the stability and accuracy of LLMs in domain-specific entity boundary detection and disambiguation [25–27]. For geography-related tasks, some methods further integrate semantic information with spatial features to improve the alignment and matching of geographic entities, thereby enhancing the consistency and usability of the entity layer within the KG [28,29]. Furthermore, the strong transferability of LLMs in NER tasks across various domains—including law, healthcare, and finance—underscores their potential to handle professional corpora characterized by complex structures and variable terminology [30–33].

Similarly, LLMs have demonstrated considerable capabilities in Relation Extraction (RE). For instance, Li et al. employed ChatGPT for zero-shot RE, achieving performance comparable to that of supervised models on several benchmarks [34]. Asada et al. improved relation identification accuracy in biomedical corpora by incorporating LLM-generated entity descriptions and causal chain explanations [35]. For more complex KE tasks, Carta et al. introduced an iterative zero-shot prompting framework for the high-quality, automatic extraction of knowledge triples from unstructured text [36], while Zhang et al. confirmed that data augmentation strategies significantly boost the triple extraction performance of LLMs in few-shot scenarios [37].

Despite their promise, applying LLMs to GeoKG construction presents several formidable challenges. First, the issue of hallucination where LLMs generate information that is inaccurate or factually incorrect poses a severe threat to the reliability of a GeoKG [38]. This problem is exacerbated during relation generation, where hallucinations can introduce fabricated facts that contaminate the knowledge base, thereby undermining its integrity and trustworthiness [39]. Second, LLMs exhibit a limited capacity to comprehend the nuanced semantics and specialized terminology within geographic documents, often lacking the depth required for precise interpretation, which can lead to extracted knowledge of insufficient granularity or bias [40]. Third, the outputs of LLMs are often unstructured and inconsistent, complicating subsequent parsing and ingestion into a structured database [41]. In multi-stage extraction pipelines, minor errors from early NER or RE stages (e.g., imprecise entity boundaries, ambiguous relation types) can be amplified during subsequent knowledge fusion, significantly degrading the overall quality and usability of the final KG [42]. These challenges are particularly acute in the geographic domain, which inherently demands high precision, spatial consistency, and logical rigor [43].

Crucially, existing frameworks have yet to effectively resolve these inherent challenges. [44,45] Most current LLM-based GeoKG methods typically rely on one-pass information extraction or standard fine-tuning pipelines. [46] While these approaches utilize the generative power of LLMs, they often treat geographic extraction as a generic NLP task, lacking the specific mechanisms to validate spatial consistency or correct logical fallacies. [47] This absence of a self-correction mechanism represents a significant gap in current methodologies, limiting their ability to ensure the high accuracy and completeness required for domain-specific applications. [48]

To address the aforementioned challenges and enhance the quality and efficiency of automated GeoKG construction, this paper proposes LLM-Reflex-GeoKG, a framework that innovatively adapts and applies the Reflexion mechanism [49] to the LLM-driven geographic knowledge extraction process. Conceptually, Reflexion functions as a verbal reinforcement learning framework. It replaces traditional weight updates with linguistic feedback, allowing the model to maintain a memory of past mistakes and iteratively refine its reasoning. Drawing inspiration from this concept, we have tailored it to the specific requirements of geographic information extraction by designing a dual-LLM collaborative architecture. In this architecture, an ’Executor’ LLM performs the initial geographic NER and RE via a sequential in-context prompting pipeline, where identified entities serve as contextual constraints for the subsequent relation extraction. Meanwhile, an “Evaluator” LLM assesses the Executor’s output against a predefined GeoKG schema, domain constraints, and the LLM’s inherent commonsense knowledge to generate structured feedback. Based on this, we have designed a bespoke optimization process for both NER and RE tasks that leverages this iterative “extract-evaluate-feedback-refine” loop(which continues until the output passes validation or reaches a predefined maximum iteration limit). Crucially, this optimization is achieved by dynamically refining the prompt context based on error feedback, rather than through parameter retraining (fine-tuning). This cycle progressively improves knowledge extraction accuracy and enhances the model’s adaptation to the target geographic domain. Furthermore, our framework integrates an entity alignment strategy that combines high-dimensional textual semantic embeddings with explicit spatial proximity constraints (calculated via geocoding and GIS spatial analysis), ensuring the quality and consistency of the entity layer in the final GeoKG. The proposed LLM-Reflex-GeoKG framework is designed to effectively resolve the key difficulties encountered when directly applying LLMs to the automated construction of high-quality, domain-specific GeoKGs.

In summary, the main contributions of this paper are threefold. First, we propose an iterative refinement framework, LLM-Reflex-GeoKG, which integrates the Reflexion mechanism for geographic knowledge graph construction. It offers a systematic solution for high-quality, automated knowledge extraction by employing a dual-LLM collaborative architecture and iterative feedback. Second, we design and implement specialized NER and RE methods optimized within this framework. Through iterative evaluation and refinement, these methods significantly improve the robustness of extraction from complex geographic texts. Finally, we demonstrate the framework’s effectiveness through experiments on a river network knowledge graph construction task, where our approach excels at populating a GIS-based spatial skeleton with semantic information, achieving superior performance compared to native LLMs.

## Materials and methods

### Research area

**Fig 1:**
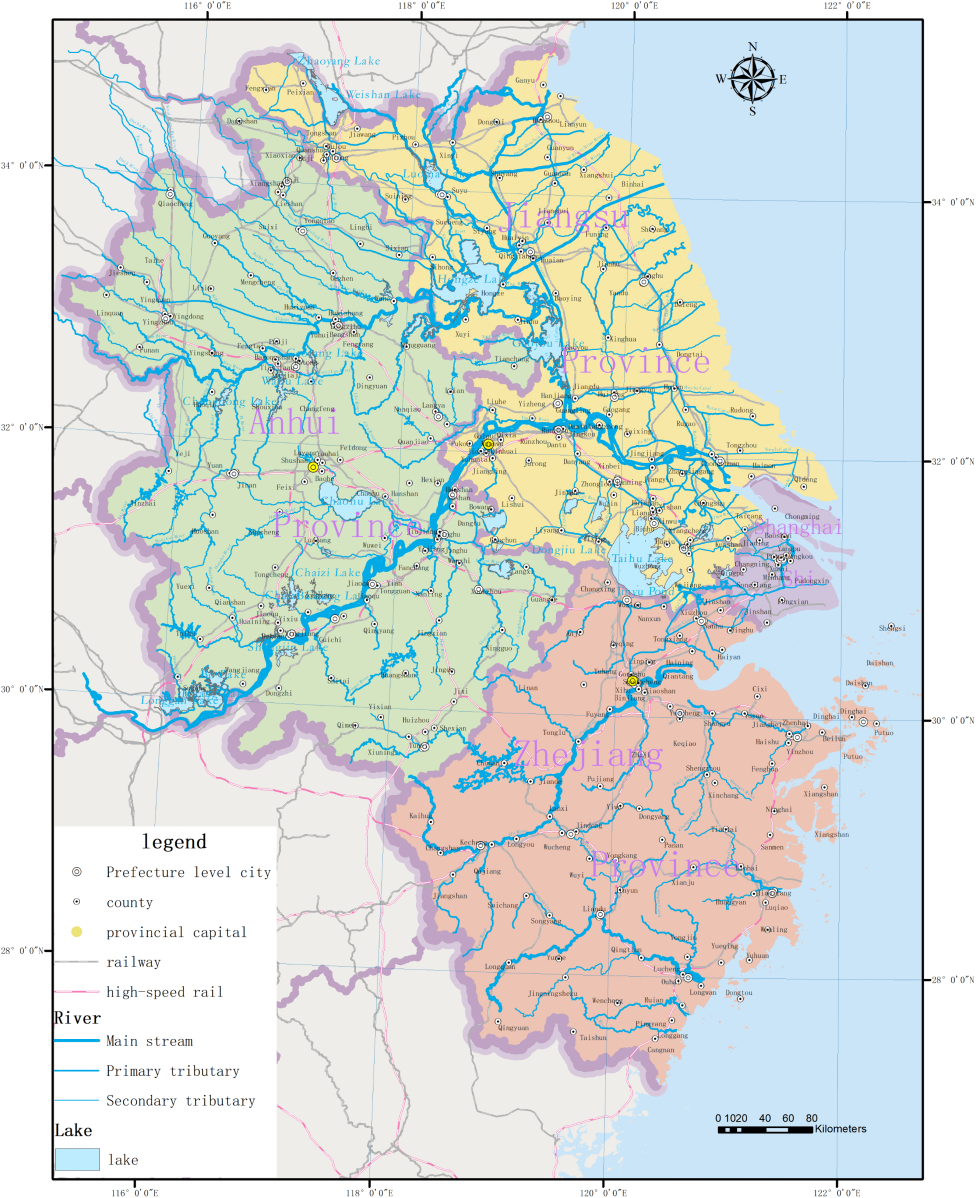
Yangtze River Delta Water Network Map, Map created using QGIS based on open data from the Standard Map Service of China (http://bzdt.ch.mnr.gov.cn/). Approval Number: GS(2020)3189.

The Yangtze River Delta (YRD) region comprises Shanghai Municipality and the provinces of Jiangsu, Zhejiang, and Anhui, covering approximately 358,000 km^2^. Geomorphologically, the region is dominated by low-lying alluvial plains with an average elevation generally below 10 meters. Climatically, it falls within the northern subtropical monsoon zone, characterized by abundant precipitation. Hydrologically, the YRD possesses one of the highest river network densities in China, encompassing complex water systems such as the Yangtze River Basin and the Taihu Lake Basin(Fig 1).

As a high-density, multi-centric urban region, the YRD presents a unique confluence of characteristics: a high diversity of geographic entity types, complex spatial relationships, and massive volumes of heterogeneous data. These conditions make the construction of a comprehensive and accurate Geographic Knowledge Graph (GeoKG) a formidable scientific challenge, necessitating advanced extraction and integration methods tailored to the region’s complexity.

### Research materials

The data basis for this study covers multi-source heterogeneous data collected extensively from the Yangtze River Delta region. The specific classification, description, content format, and source examples are shown in Table 1. The Description column describes specific data items, the Content column describes the data format or information type of each data item, and the Source column describes the source of the data items. The original data is described in Chinese.

**Table 1:** Yangtze river delta data categories.

Main data category	Specific type	Description	Content	Sources
Structured Data	Geospatial Vector Data	Administrative boundaries, water network data (rivers, lakes).	Points, lines, polygons and their attributes.	National Geospatial Information Center (NGCC).
	Geospatial Raster Data	Digital Elevation Model (DEM) representing terrain elevation.	TIF	Copernicus Open Access Hub (ESA).
	Statistical Data	Structured tabular data on various aspects of administrative units in the Yangtze River Delta region.	Numeric, text.	Official releases from the National Bureau of Statistics and Statistical Bureaus of Shanghai, Jiangsu, Zhejiang, and Anhui.
Structured Data	Structured Knowledge Entries	Digitized encyclopedic entries on Jiangnan culture and the Yangtze River Delta region.	Text	Encyclopedia of Jiangnan Culture and supplementary online encyclopedias.
Semi-structured Data	Plain Text Data	Plain text content from a variety of sources.	Text	Encyclopedia of Jiangnan Culture, academic literature databases, and supplementary online encyclopedias.

### Geographic data collection and processing

Data collection for this study focused on core geographic elements within the Yangtze River Delta region, including vector datasets of rivers, lakes, and cities (represented by administrative divisions). These geographic datasets were obtained from the National Geospatial Information Center (NGCC), which provides standardized and officially curated national fundamental geographic information. Digital Elevation Models (DEM) from the Copernicus Open Access Hub (ESA) were incorporated for validation and supplementary analyses.

The data processing workflow commenced with the unification of all geospatial datasets under a single Coordinate Reference System (CRS) to ensure analytical accuracy. Subsequently, the vector datasets (rivers, lakes, and cities) were subjected to rigorous topological validation and correction to resolve geometric errors, while their corresponding attribute information was systematically cleaned and standardized.

River datasets, a central focus of this research, underwent a specialized classification and encoding procedure. We employed a hybrid approach, initially classifying and encoding rivers according to the Chinese national standard SL 249–2012. To account for smaller tributaries not covered by this standard, we applied the classic Strahler stream order method. This method assigns an order to each stream segment by analyzing the network topology, starting from the headwaters. Specifically, headwater streams with no tributaries are designated as first-order. The stream order increases at confluences: when two streams of the same order n merge, the resulting downstream segment is elevated to order n + 1. Conversely, when streams of different orders meet, the downstream segment simply retains the higher of the two orders. This dual-method approach enabled the classification of the YRD river system into a distinct hierarchy—comprising main channels, primary tributaries, and secondary tributaries—and the assignment of order and code information to each segment. This detailed codification provides a robust foundation for analyzing the network’s hierarchical structure and connectivity patterns.

Finally, lake and city datasets underwent boundary calibration and attribute standardization to ensure spatial consistency with other geographic features. The DEM raster data were also preprocessed to align their coordinates and spatial resolution with the vector layers. All processed datasets were ultimately integrated to form standardized geographic layers, establishing a spatial data foundation characterized by strict topological consistency (specifically ensuring river network connectivity and correct flow directionality) for the subsequent construction of the Geographic Knowledge Graph.

### Text and attribute data collection and processing

Building upon the processed geospatial data, we compiled a multi-source collection of textual and attribute data pertaining to the rivers, lakes, and cities of the YRD. The objective was to enrich the knowledge graph with deep semantic context and background knowledge. Given the diversity of the sources, tailored processing workflows were developed for each data type.

Textual data were compiled from a multi-source collection, primarily the authoritative Encyclopedia of Jiangnan Culture [50], supplemented by historical documents, news archives, descriptive sections of statistical yearbooks, and online encyclopedias (e.g., Baidu Baike). It is important to note that open-source data such as Baidu Baike served strictly as supplementary references for semantic richness and underwent manual cross-verification against authoritative records to ensure accuracy. The temporal scope of the data collection was unified to the year 2021 to ensure consistency with the river network spatial dataset. The preprocessing pipeline began with the extraction of plaintext from various source formats, employing standard Optical Character Recognition (OCR) tools for image-based documents, followed by manual inspection to ensure text legibility. This was followed by a rigorous cleaning phase to eliminate specific noise types, including HTML tags, special characters, garbled text, and irrelevant headers/footers. In the final, critical step for compatibility with LLMs, the cleaned text was processed through text chunking—segmenting it into semantically coherent units (e.g., sentences or paragraphs)—to prepare it for subsequent named entity recognition and relation extraction.

The acquisition of attribute data prioritized structured and semi-structured information. Key sources included multi-dimensional tabular data from statistical yearbooks and standardized properties, such as river classifications and codes, from official technical standards. Additionally, some attributes were derived directly from geospatial measurements. Preprocessing for all attribute data involved format standardization, unit unification(e.g., normalizing lengths to kilometers), code alignment(e.g., matching administrative codes to the GB/T 2260 national standard), and establishing feature-level attribute linkage to their corresponding geospatial entities.

This comprehensive workflow yielded a fully populated dataset covering all 173 rivers identified in the spatial skeleton, where each record is intrinsically linked to a specific geospatial entity in the YRD. These datasets, in conjunction with the geospatial layers from the preceding stage, serve as the primary input for the automated construction of the Geographic Knowledge Graph using our proposed LLM-based Reflexion framework.

## Methods

**Fig 2:**
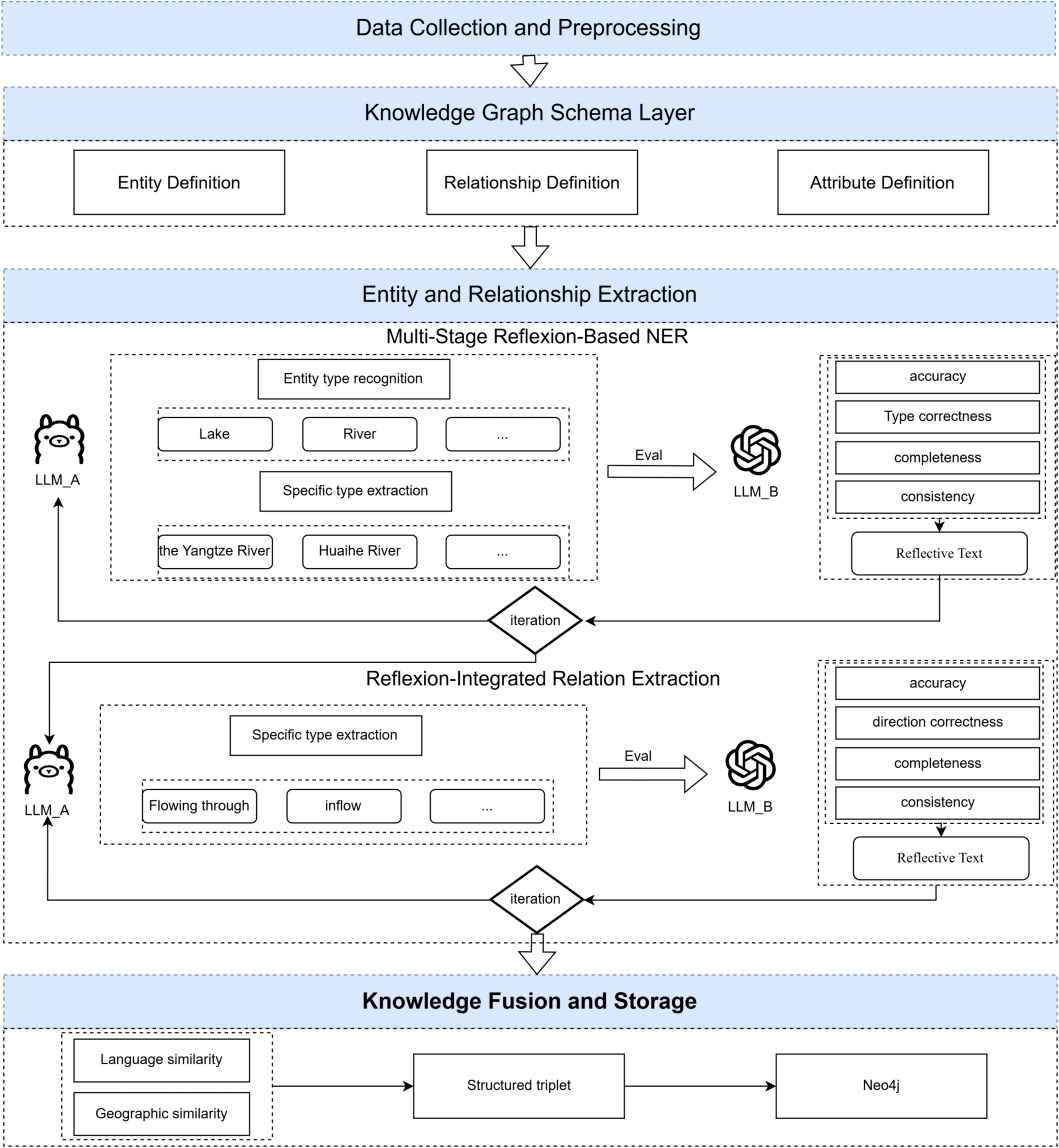
The overall process of knowledge graph construction.

A geographic information knowledge graph aims to structurally describe geographic entities, concepts, events, and their spatio-temporal and semantic relationships in the physical world. Its basic units are typically triples consisting of (geographic entity-relationship-geographic entity) or (geographic entity-attribute-attribute value). The overall framework for constructing a geographic information knowledge graph that integrates the Reflexion mechanism is shown in Fig 2.

### Knowledge graph schema definition

**Fig 3:**
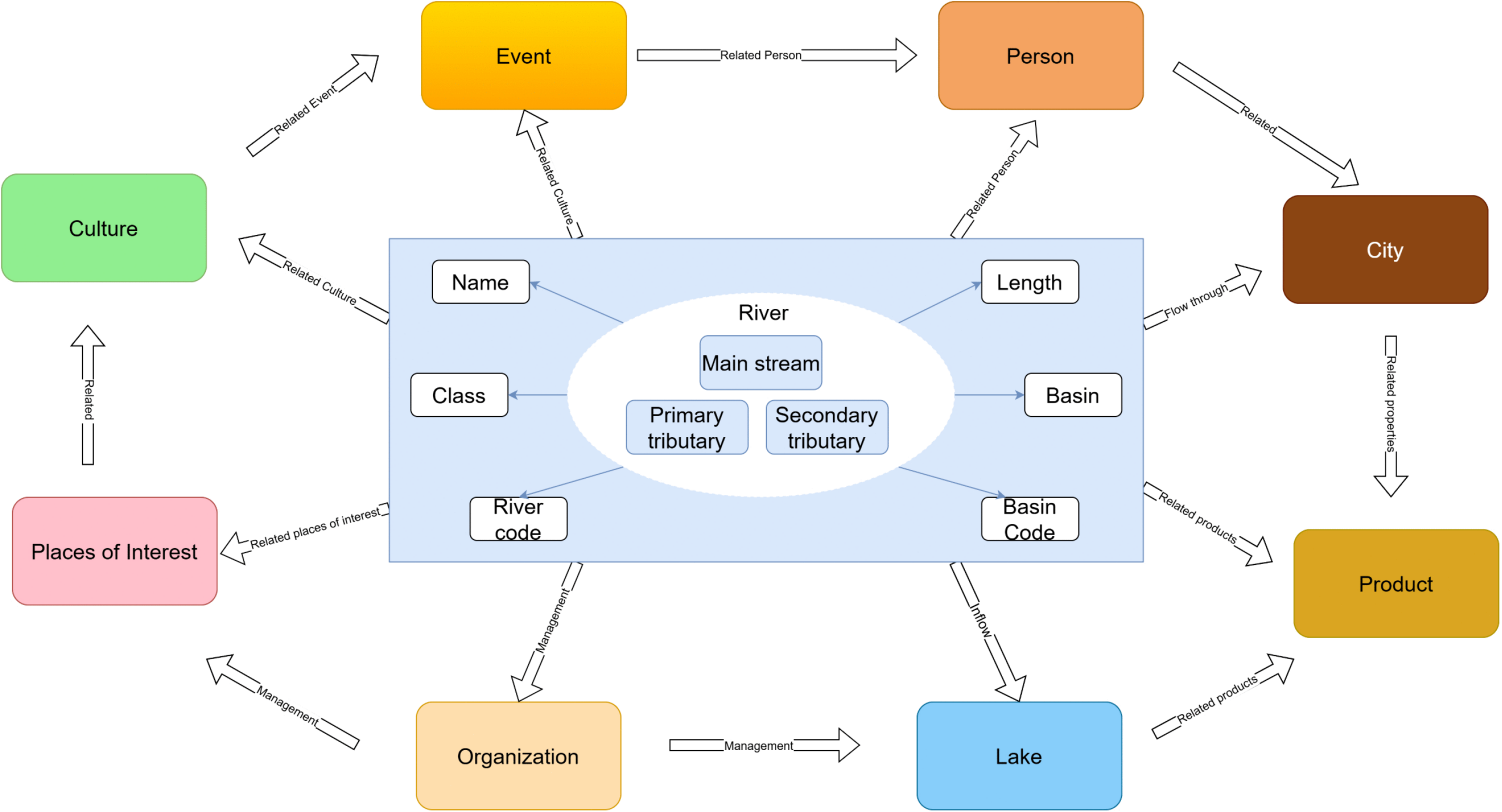
Schema diagram.

The knowledge graph schema formally defines the data structures, providing the foundational blueprint for subsequent knowledge extraction, storage, and application. For this study, we designed a schema tailored to the geographic characteristics of the Yangtze River Delta,as illustrated in Fig 3. Unlike discrete spatial data that relies heavily on static attributes, the water network’s semantic richness is encoded primarily through its connectivity and relational structure. To capture this continuous and structured nature, our schema prioritizes hierarchical entity types and topological relation types, supplemented by essential descriptive attributes.

Entity Types were defined to map the core geographic objects and relevant phenomena within the YRD. To rigorously reflect the unique topological structure of the water network, the River entity was explicitly sub-categorized into Main stream, Primary tributary, and Secondary tributary. This hierarchy aligns with the classification methodology detailed in Section 3.2, which integrates the national standard (SL 249–2012) with the Strahler stream order method. Additionally, we predefined a set of domain-specific entity types—including Lake, City, Event, Person, Product, Location, Organization, Purpose and Function, and Culture. To maintain semantic coherence, the scope of these non-geographic entities is strictly constrained to instances with direct semantic or spatial dependencies on the water network. These definitions serve as the standardized target categories for the subsequent knowledge extraction process.

Relation Types define the diverse semantic links and spatial interactions among the predefined entities. These relations are critical for guiding the LLM-based knowledge extraction process and for building a networked knowledge structure. We classify these relations into two principal categories: spatial and non-spatial. Spatial relations are derived from Geographic Information System (GIS) operations and are subsequently refined through domain-specific rules and inference to imbue them with semantic meaning, as detailed in Table 2. To ensure interoperability and reusability, these relations are semantically mapped to standard OGC GeoSPARQL properties (e.g., Flows through aligns with geo:sfIntersects, Adjacent aligns with geo:sfTouches).

**Table 2:** Spatial relations and dependent GIS operations.

Relation Name	Domain	Range	Dependent GIS Ops.	Description
Flows through city	River	City	Intersects, Crosses, Within	Refers to a river running through the administrative area of a particular city.
Involves river	Lake	River	Intersects, Within/Contains	Refers to the spatial association of an entity with a river.
Located in	Location; City; Event; Organization	City; Location	Within, Contains	Refers to an entity being within another entity or in a specific geographical location.
Adjacent	City; Lake; Location	City; Lake; Location	Touches	Refers to two geographic entities that spatially border or share a boundary with each other.
Inflow	River	Lake; River	Intersects; Flow Direction	Refers to the confluence of one body of water (e.g., a river) into another body of water.
Superior river	River	River	Intersects; Flow Direction	Refers to a river that is the upper main stem or main channel of another river.
Tributary	River	River	Intersects; Flow Direction	Refers to a smaller stream that feeds into another stream of higher grade or higher flow.

Non-spatial relations are employed to articulate the rich semantic context surrounding geographic entities. They go beyond physical topology to encode conceptual hierarchies (e.g., subclass-of), dynamic event associations, and the intricate socio-economic or cultural dependencies that link the natural water system to human activities.The definitions and descriptions of these non-spatial relations are provided in Table 3.

**Table 3:** Non‐spatial relations and definitions.

Relation Name	Domain	Range	Description
Flow through	Location, City	Location, City	Describes the area or specific landmarks through which the river flows, without focusing on precise topology.
Also known as	Location, City River, Lake Organization	Location, City River, Lake Organization	Connecting entities with their aliases or historical names.
Originates from	River, Event	Location, City	Indicates where or why a river, event, etc. originated.
Manages	Organization	City, Event; River, Lake	Indicates the agency’s authority or responsibility for the management or governance of areas, rivers, events, etc.
Related person	Event, Culture Location, City River, Lake Organization	Person	Connects an entity to people who have a significant impact on or connection with it.
Purpose and Function	River, Lake Organization	Purpose and Function (Entity)	Describes the primary use or socioeconomic function of a geographic entity or a specific area.
Involves region	Event Organization Product	Location, City	Denotes an area to which an event, plan, or entity is thematically or logically related, without emphasis on precise spatial containment.
Related event	Location, City River, Lake Person Organization	Event	Connects an entity to the events that occur or take place with it.
Related produce	Location, City River, Lake	Produce	Connecting a region or specific geographic environment with its unique natural resources or products.
Related place of interest	Location, City River	Location	Connects a larger geographical entity with the ancient towns, streets, etc., with historical and cultural significance within or around it.
Related culture	Location, City Event Place of interest	Culture	Connecting geographical entities with related cultural phenomena, traditions, customs, or intangible cultural heritage.
Related to	All entity types	All entity types	Indicates that there is some undefined connection between two entities.

### Multi-stage geographic entity recognition integrating the Reflexion

Geographic Named Entity Recognition (NER) is a foundational task in the construction of Geographic Knowledge Graphs (GeoKGs), involving the accurate and comprehensive identification of predefined geographic entities and their corresponding categories from large-scale text corpora. To address this task, this study proposes a multi-stage Geographic NER method leveraging a LLM augmented with the Reflexion mechanism. The workflow of this method comprises three primary stages: Initial Entity Extraction, Reflexion-based Evaluation and Feedback, and Iterative Refinement and Entity Confirmation.

#### (1) Initial entity extraction with LLM_A.

The primary objective of this initial stage is to employ an extraction model (LLM_A) to automatically identify and extract candidate geographic entities from the preprocessed text data. To enhance the performance of LLMs on this task, we introduce a multi-stage recognition strategy. This strategy employs a coarse-to-fine approach, first identifying the potential entity types within the text before proceeding to extract the specific instances for each identified type. This prevents the model from conducting unconstrained searches under broad instructions, thereby improving both efficiency and precision. This strategy comprises two core sub-steps, which are performed via separate, sequential LLM calls to ensure precise instruction adherence:

- **Entity Type Scoping** In this step, for each input text unit, a specialized prompt is constructed to guide LLM_A in analyzing the text. The model is tasked with identifying a likely subset of entity types—from the full list defined in the knowledge graph schema—that may be present in the unit. This procedure narrows the search space for the subsequent extraction phase, enhancing its focus and overall efficiency.
- **Targeted Entity Instance Extraction** Subsequently, for each entity type identified during the scoping step, LLM_A receives a new, more targeted prompt. This prompt, containing both the original text and the specific target type, instructs the model to identify and extract all explicit instances of that entity from the text. This extraction process iterates strictly through the identified type list and terminates automatically once all types have been processed, effectively preventing redundant or open-ended model interactions.

#### (2) Reflexion mechanism: Entity evaluation and feedback.

This stage introduces an independent LLM, designated as the Evaluator (LLM_B), to critically appraise the candidate entities extracted by LLM_A. Distinct from prior reflexive pipelines that typically rely on single-agent self-correction or binary feedback signals, our geographic implementation adopts a collaborative dual-LLM architecture. In this setup, the Evaluator specifically scrutinizes the output for spatial inconsistencies (e.g., topological conflicts) and semantic errors defined by the GeoKG schema. Crucially, it provides feedback in the form of structured ’Reflective Text’—natural language explanations that explicitly guide the Executor on how to rectify these domain-specific errors in the subsequent iteration.

#### (3) Iterative refinement and entity confirmation.

Upon receiving the Reflective Text from the Evaluator (LLM_B), the extraction model (LLM_A) begins the iterative refinement process. The objective is to leverage the insightful feedback from LLM_B to make targeted improvements to the initial extraction results. This iterative process progressively enhances the accuracy and recall of the geographic NER, leading to the confirmation of a high-quality entity set. The feedback from LLM_B is inserted directly into the prompt context for LLM_A’s subsequent attempt on the same text unit. Unlike long-term archival memory, this feedback serves as immediate, transient context to guide the model’s reasoning and extraction strategy for the current iteration. LLM_A then adjusts its extraction strategy to generate a more accurate list of candidate entities.

This “Extraction (LLM_A) → Evaluation (LLM_B) → Refined Extraction (LLM_A)” loop can be repeated until a predefined maximum number of iterations is reached. In this manner, the Reflexion mechanism establishes an effective “continuous learning” cycle for geographic NER, without requiring any parameter fine-tuning of the LLMs themselves.

### Relation extraction with reflexion

Elucidating the relationships between geographic entities is as crucial as identifying the entities themselves. In this study, these relationships are categorized as either spatial or non-spatial. While spatial relations are programmatically derived via GIS analysis (as detailed in our Knowledge Graph Schema), this section focuses on a method for automatically extracting predefined non-spatial relations from text. This process, which integrates the Reflexion mechanism, also comprises three core stages: preliminary relation extraction, Reflexion-based evaluation and feedback, and iterative refinement and confirmation.

#### (1) Initial relation extraction with LLM_A.

Following the identification of high-quality geographic entities, the system first generates candidate entity pairs for each text unit containing at least two such entities. Subsequently, for each candidate pair, a tailored prompt is constructed to guide the extraction model (LLM_A). This prompt integrates the original text, the candidate entity pair (including entity types), and the predefined list of non-spatial relation types. LLM_A is then tasked with analyzing the contextual semantic links between the pair to determine if a predefined relation exists and, if so, to select the most appropriate one. The output of this stage is a set of candidate non-spatial relation triples.To prevent overgeneralization in conceptually broad categories (e.g., ’Related person’ or ’Related culture’), we incorporated strict inclusion criteria into the prompt instructions. Specifically, the model was tasked to extract relations only when the source text provided explicit evidence of a direct and significant influence (e.g., historical governance, cultural origin, or artistic depiction), while tangential or weak associations were explicitly filtered out via negative examples.

#### (2) Reflexion-based evaluation and feedback.

This stage employs an independent Evaluator model (LLM_B) to rigorously validate the initial extraction results. The evaluation process synthesizes multiple information sources: the original text’s context, confirmed entity details (to verify adherence to relation-specific domain and range constraints), the definitions of non-spatial relation types, and general commonsense knowledge for logical consistency checks. Its core task is to pinpoint extraction deviations, such as incorrect relation classifications, reversed relation directions, domain-range mismatches, or the extraction of spurious relations not supported by the text. Following this analysis, LLM_B generates a structured “Reflective Text” that not only identifies errors but also provides causal explanations and actionable suggestions for correction.Crucially, this mechanism transforms rigid graph schema constraints into interpretable linguistic guidance, enabling the model to perform semantic debugging on geographic logic rather than merely optimizing for textual probability.

#### (3) Iterative refinement and relation confirmation.

The extraction model leverages the Reflective Text from LLM_B to perform targeted self-correction. For subsequent extraction attempts on the same entity pair, this feedback is explicitly incorporated into the new prompt for LLM_A, serving as critical context to guide its reasoning and extraction strategy.

As with the entity recognition task, this iterative loop can be repeated until the quality of the extracted relations meets predefined standards or a maximum number of iterations is reached. This internal, language-based feedback mechanism effectively enhances the model’s relation extraction performance on complex texts without requiring parameter fine-tuning. The non-spatial relation triples validated through this iterative process exhibit superior accuracy and reliability, forming the critical semantic links within the knowledge graph.

### Knowledge fusion and storage

Upon completing the stages of entity recognition, spatial relation computation, and non-spatial relation extraction, the output consists of numerous structured geographic knowledge fragments. The subsequent phase involves knowledge fusion and storage, which are essential for constructing a unified, consistent, and high-quality Geographic Knowledge Graph (GeoKG). Knowledge fusion is a critical process for ensuring consistency and eliminating redundancy within the graph. Its core challenge is entity alignment—the task of identifying and linking different textual mentions that refer to the same real-world object.

To address this, our study employs a hybrid entity alignment strategy that integrates both semantic and geospatial similarity metrics. The final alignment score, $S(e_{i},e_{j})$, is produced by a weighted combination of these two components. The semantic component is quantified using cosine similarity on entity embeddings from the BGE M3-Embedding model, while the geospatial component is derived from the physical distance via a Gaussian kernel function. This is formally expressed as:


$$S(e_{i},e_{j})=\underset{\text{Semantic Similarity}}{\underbrace{\alpha \cdot \frac{𝐀\cdot 𝐁}{\left\|𝐀\|\|𝐁\right\|}}}+\underset{\text{Geospatial Similarity}}{\underbrace{(1-\alpha )\cdot \exp\left(-{\left(\frac{d}{\sigma }\right)}^{2}\right)}}$$ (1)

where **A** and **B** represent the *n*-dimensional embeddings for entities *e*_*i*_ and *e*_*j*_, *d* is the geospatial distance between them, α∈[0,1] is a weight parameter, and σ is a tunable scaling parameter for the Gaussian kernel.

An entity pair was identified as coreferent if its alignment score $S(e_{i},e_{j})$ exceeded a predefined thresholds, which was set to 0.85 in this study.This value was determined empirically to prioritize high precision, ensuring that distinct geographic entities are not incorrectly merged. The identified coreferent mentions are subsequently merged and assigned a unified global identifier. Furthermore, a source-priority heuristic was established to resolve knowledge conflicts, particularly inconsistencies between textual knowledge and GIS-derived data. Specifically, topological relations derived from quantitative GIS analysis were assigned absolute precedence, serving as the spatial ground truth; consequently, any text-extracted relation that contradicts these topological constraints is automatically discarded to preserve the geospatial integrity of the GeoKG

Upon completion of the entity alignment and relation validation processes, the final, structured knowledge was organized into (subject, predicate, object) triples. For persistence and management, the Neo4j graph database was selected, as its native graph structure is well-suited to the complex and highly interconnected nature of the GeoKG.

## Results

### Experimental setup

#### Dataset and evaluation metrics.

To quantitatively evaluate the performance of our proposed framework, we constructed a high-quality, annotated corpus from the multi-source, heterogeneous data described in Section 2.1. The corpus was developed using a human-in-the-loop workflow meticulously guided by domain experts. This process involved an initial phase where experts manually annotated a seed dataset to establish clear guidelines, followed by a pre-annotation step where a LLM processed the remaining data. In the final stage, all annotations underwent a unified expert review and correction. This methodology significantly enhanced efficiency while ensuring the reliability of the results through a single, consistent professional standard. The final corpus comprises approximately 700 text fragments (totaling over 80,000 Chinese characters). We acknowledge that the dataset size is moderate in terms of raw count; however, the fragments were carefully curated to ensure typological diversity, covering a wide spectrum of geographic entities (e.g., rivers, lakes, cities) and relation types characteristic of the region. These include both spatial relations (e.g., ’Inflow’, ’Adjacency’) and semantic relations (e.g., ’Economic function’, ’Cultural attribute’), ensuring the dataset effectively reflects the complexity of the Yangtze River Delta water network. This corpus serves as the benchmark for objectively measuring the performance of our knowledge extraction method in both zero-shot and few-shot scenarios.

The efficacy of the proposed LLM-Reflex-GeoKG framework was comprehensively validated through a systematic experimental design. Our evaluation strategy was twofold: first, to establish the overall high performance and practical value of the complete framework on geographic information extraction tasks; and second, to quantitatively analyze the individual contributions of its innovative components, such as the multi-stage strategy and the Reflexion mechanism, via rigorous ablation studies. This dual-pronged evaluation not only confirms the framework’s effectiveness but also elucidates the underlying drivers of its performance, providing robust empirical support for our methodological innovations.

To evaluate the effectiveness of the proposed method in identifying and extracting knowledge from text, we adopt three standard metrics used in information extraction—precision, recall, and F1 score—to assess its performance across different tasks. The calculations for these metrics are:


$$Precision=\;\frac{TP}{TP+FP}$$ (2)

$$Recall=\frac{TP}{TP+FN}$$ (3)

$$\text{F1-score}=2\cdot \frac{\text{Precision}\cdot \text{Recall}}{\text{Precision}+\text{Recall}}$$ (4)

where TP, FP, and FN respectively denote true positive (correctly predicted positive samples), false positive (incorrectly predicted positive samples), and false negative (incorrectly predicted negative samples).

Take the NER results as an example. These results contain numerous predicted entity-label pairs. Precision and recall reflect the overall quality of the constructed GeoKG from two different aspects—namely, how accurately the entities are predicted and how completely the relevant entities are captured. The F1 score, as their harmonic mean, provides a balanced summary; we report micro-averaged results across all entity types under exact-match evaluation.

### Implementation details

All models were accessed via their standard API endpoints. Our framework employs an asymmetric dual-LLM architecture designed to optimize both efficiency and reasoning depth. The Extractor model is powered by DeepSeek-V3, selected for its high throughput and strong instruction-following capabilities. Since the extraction phase involves processing large volumes of text, V3 ensures computational efficiency. Conversely, the critical role of the Evaluator is fulfilled by DeepSeek-R1, a model optimized for complex reasoning tasks. R1’s superior Chain-of-Thought (CoT) capabilities allow it to perform deep logical verification, ensuring that the generated ’Reflective Text’ provides insightful feedback.

In few-shot configurations, prompts were augmented with five curated examples for each task. These examples were strictly excluded from the test corpus to ensure zero data leakage. To minimize generation randomness, we set the temperature to 0.1, while all other hyperparameters were maintained at their standard API default values.The Reflexion process was configured for a maximum of five iterations, with an early-stopping condition triggered by two consecutive rounds of identical results.This limit was informed by empirical patterns in iterative refinement [49], where performance gains typically plateau after the initial few cycles, allowing us to balance extraction quality with computational costs.

To systematically assess the contribution of each core component within our framework, we designed a series of ablation studies for both the NER and RE tasks.

For the Geographic NER task, we established a set of comparative configurations. We began with a baseline Zero-shot model (LLM_A) to measure its raw capability. To assess the impact of examples, a Single-stage Few-shot configuration was introduced. Building upon this, to validate our multi-stage strategy, we implemented a Two-stage Few-shot method, where LLA_A first performs entity type scoping before conducting targeted instance extraction. Finally, to quantify the efficacy of the Reflexion mechanism, it was integrated into both setups, creating the Single-stage + Reflexion and the Two-stage Few-shot + Reflexion configurations, with the latter representing our full NER method.

Similarly, for the non-spatial relation extraction task, four configurations were designed: (1) a Zero-shot setup using only LLM_A; (2) a Few-shot setup including annotated examples in the prompt; (3) a Zero-shot + Reflexion setup, which augments the zero-shot approach with the evaluation-feedback loop; and (4) a Few-shot + Reflexion setup, which combines both techniques and constitutes our full relation extraction method. Detailed results and analysis for these experiments are presented in the subsequent sections.

### Geographic entity recognition results

This section evaluates the performance of our proposed multi-stage method for Geographic Named Entity Recognition (NER). We employed Precision, Recall, and F1-score as the standard evaluation metrics, with experiments conducted on our custom-built annotated corpus for the Yangtze River Delta. To analyze the contribution of each component within our framework, we designed a series of ablation studies comparing zero-shot versus few-shot learning, single-stage versus two-stage extraction, and the inclusion versus exclusion of the Reflexion mechanism. The comprehensive results are presented in Table 4.

**Table 4:** Performance comparison of different NER methods.

Method	Precision	Recall	F1-score
Single-stage Zero-shot	0.809	0.805	0.807
Single-stage Few-shot	0.825	0.816	0.820
Two-stage Few-shot	0.836	0.867	0.852
Single-stage + Reflexion	0.858	0.886	0.872
Two-stage Few-shot + Reflexion	0.904	0.893	0.898

The results in Table 4 provide direct quantitative evidence for the efficacy of each strategy within our proposed framework. An analysis of the overall trends reveals several key findings. While the single-stage few-shot method shows a slight improvement over its zero-shot counterpart across all metrics, the gains are marginal, suggesting that the addition of only a few examples has a limited impact. In contrast, adopting the two-stage few-shot method yields a substantial performance increase. Notably, Recall improves from 0.816 to 0.867, boosting the F1-score to 0.852. This indicates that the addition of a fine-grained, secondary recognition step effectively addresses the issue of missed entities (false negatives) common in single-stage approaches, thereby enhancing the overall comprehensiveness of the NER task.

Furthermore, the integration of the Reflexion mechanism provides an even more significant performance boost. For instance, when applied to the single-stage method, Reflexion elevates Precision to 0.858 and Recall to 0.886, a clear advantage over the baseline single-stage approach. This demonstrates that by dynamically analyzing and correcting errors, the Reflexion mechanism enables the model to more accurately re-evaluate and classify ambiguous or easily confused geographic entities.The substantial net improvement in overall F1 scores serves as the primary quantitative validation of the Evaluator’s efficacy in identifying and guiding the correction of domain-specific errors.

Crucially, our full proposed method—the Two-stage Few-shot + Reflexion configuration—achieved the best performance, with a Precision of 0.9036, a Recall of 0.8926, and a final F1-score of 0.898. This result significantly outperforms the standard two-stage few-shot method, highlighting how the synergistic integration of a multi-stage strategy with the Reflexion mechanism can effectively mitigate error propagation. This combination enhances both the precision and the stability of the geographic entity recognition process.

In summary, these results demonstrate that our proposed framework effectively synergizes the fine-grained advantages of a multi-stage design with the dynamic error-correction capabilities of the Reflexion mechanism, leading to a significant and robust improvement in performance on the Geographic NER task.

### Relation extraction results

This section evaluates the performance of our proposed framework on the task of non-spatial relation extraction (RE). The experiments were conducted on our custom-built annotated corpus, using Precision, Recall, and F1-score as the evaluation metrics. To validate the efficacy of few-shot learning and the Reflexion mechanism, we compared the performance of four distinct model configurations.

The experimental results, summarized in Table 5, provide a quantitative basis for evaluating the effectiveness of each strategy. Under the Zero-shot condition, the baseline model exhibited limited performance, achieving an F1-score of only 0.5534 (Precision: 0.6321, Recall: 0.4921). This indicates that without explicit examples, the model’s intrinsic ability to identify geographic relations is constrained. However, the introduction of the Reflexion mechanism (Zero-shot + Reflexion) yielded a substantial improvement, boosting the F1-score to 0.7332—a relative increase of approximately 32.5%. This gain is attributed to balanced improvements in both Precision (0.7704) and Recall (0.6995), demonstrating that the mechanism effectively enables the model to self-correct extraction errors.

**Table 5:** Performance comparison of different RE methods.

Method	Precision	Recall	F1-score
Zero-shot	0.632	0.492	0.553
Zero-shot + Reflexion	0.770	0.700	0.733
Few-shot	0.755	0.862	0.805
Few-shot + Reflexion	0.813	0.847	0.830

Separately, the Few-shot configuration also produced a significant performance uplift, reaching an F1-score of 0.8048 (a 45.4% improvement over the zero-shot baseline), driven primarily by a surge in Recall to 0.8618. This highlights the effectiveness of in-context examples for enhancing the model’s recognition capabilities. Notably, the Reflexion mechanism demonstrates a powerful bootstrapping capability in the zero-shot setting; through iterative evaluation and linguistic feedback, it guides the model to learn and refine its extraction behavior, akin to a process of internal example generation.

The optimal overall performance was achieved when combining few-shot learning with the Reflexion mechanism (Few-shot + Reflexion), our framework’s full implementation for RE. This configuration reached an F1-score of 0.8296, a 3.1% relative improvement over the standard few-shot method. This gain was primarily driven by a significant increase in Precision to 0.8132 (a 7.7% relative increase), accompanied by a slight decrease in Recall to 0.8467. This phenomenon suggests that the Reflexion mechanism acts as a crucial refinement filter, enhancing the overall quality and precision of the extracted relations by reducing false positives, even at the cost of being more conservative with ambiguous, borderline cases.

In conclusion, the results systematically demonstrate the efficacy of our Reflexion-augmented approach for non-spatial relation extraction. The framework proves particularly effective at enhancing precision and mitigating error propagation, offering a robust solution to the challenges of data sparsity and high complexity inherent in relation extraction tasks within the geographic information domain.

### Structure and application of the river network KG

**Fig 4:**
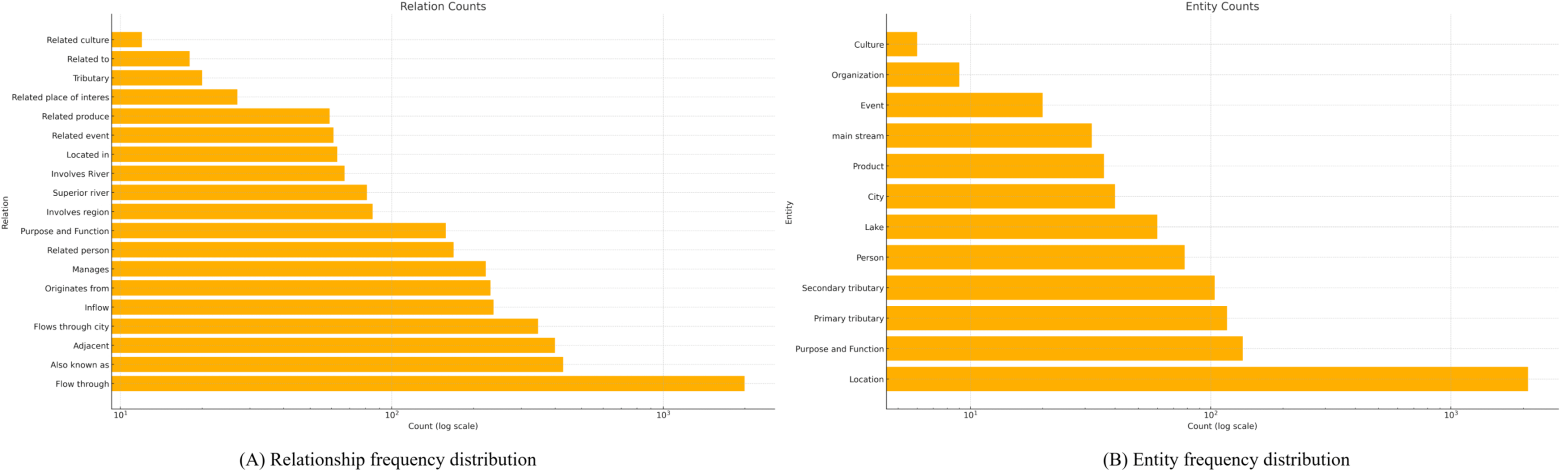
Overall size of the knowledge graph. **(A)** Relationship frequency distribution. **(B)** Entity frequency distribution.

**Fig 5:**
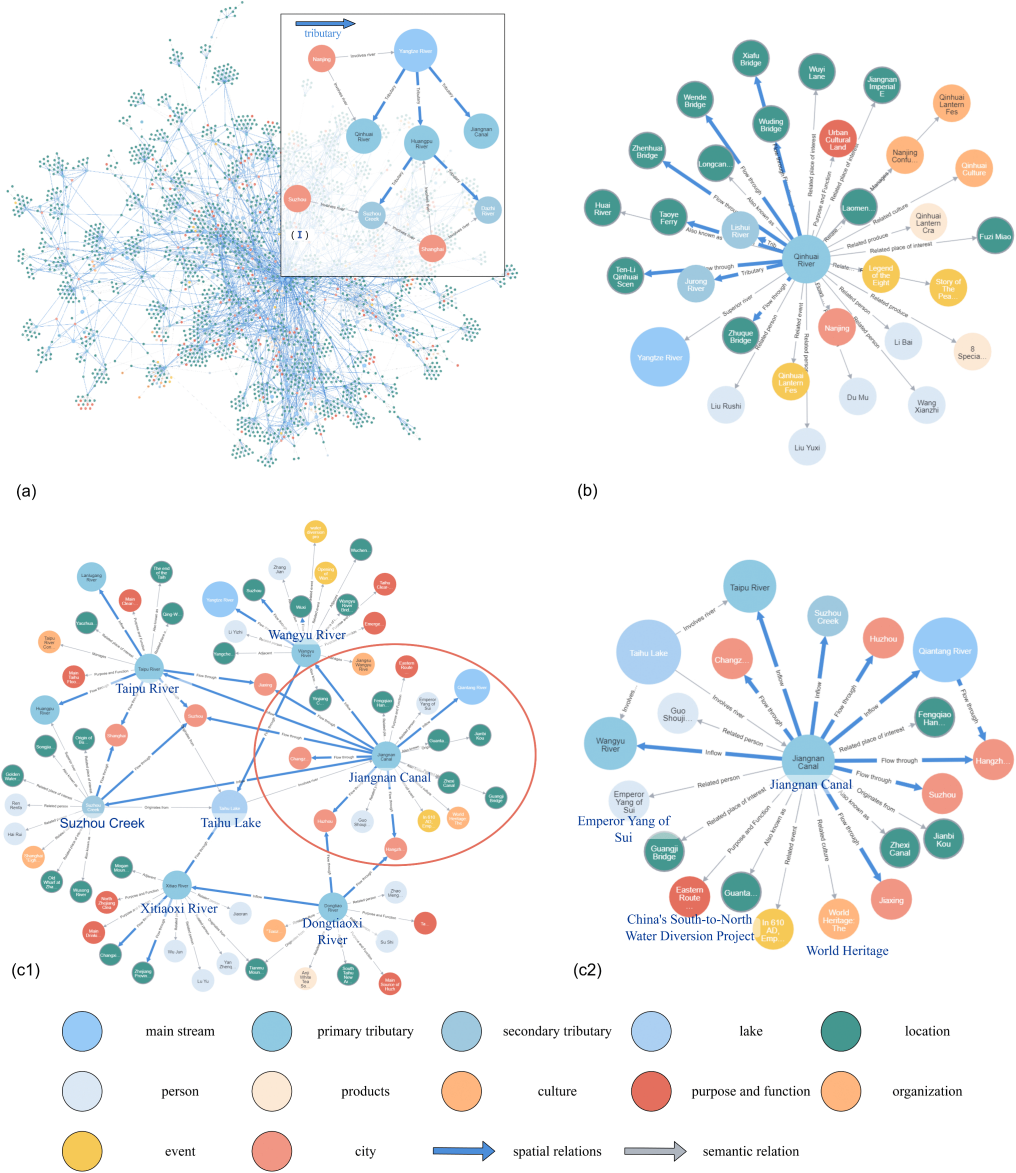
Overview of the Yangtze river delta knowledge graph structure.

The overall scale of the constructed Yangtze River Delta Geographic Knowledge Graph (GeoKG) is detailed in Fig 4. This figure illustrates the frequency distributions for both the relations (Fig 4(A)) and the entities (Fig 4(B)) within the graph. The resulting GeoKG exhibits a complex, composite knowledge structure. To qualitatively analyze this complex structure and demonstrate its capabilities, Fig 5 provides a multi-panel visualization detailing the graph’s foundational backbone, semantic enrichment, and application potential

Fig 5(a) illustrates the foundational spatial backbone of our GeoKG. This backbone is formed by the main stream (Yangtze River), primary tributaries (e.g., Qinhuai River, Huangpu River), and secondary tributaries (e.g., Suzhou Creek), which are solidly connected by geospatially-precise spatial relations (thick blue arrows) to form a hierarchical river network covering the Yangtze River Delta. The accuracy of this GIS-derived backbone is of paramount importance, as it provides the non-negotiable geographic integrity upon which all subsequent semantic applications are built.

Fig 5(b) provides a methodological deep-dive centered on the Qinhuai River, which qualitatively illustrates the semantic enrichment achieved by our LLM-Reflex-GeoKG framework. This panel clearly demonstrates the hybrid nature of the graph, showing how a single entity is simultaneously defined by two distinct relationship types, as depicted in the legend. It retains its foundational spatial relations (thick blue arrows), such as its connection to the Yangtze River. Concurrently, it is enriched by a dense web of framework-validated semantic relations (thin gray arrows). The high fidelity of this enrichment, visually demonstrated here, corroborates the strong quantitative performance reported in Table 4 and Table 5, showcasing the framework’s ability to connect the river to a multi-dimensional array of knowledge, such as historical figures (Li Bai), cultural landmarks (Nanjing Fuzimiao), and related events (Qinhuai Lantern Fair).

Fig 5(c) demonstrates the graph’s application potential for complex knowledge discovery. Leveraging the river network as the connective backbone of the entire Yangtze River Delta, our framework enables users to trace and query knowledge across this vast region. This capability is demonstrated by focusing on a pivotal hub in the network: the Taihu Lake Basin. Figure 5(c1) visualizes the result of this broad regional query, serving as a visual index of the complex spatial (spatial relations) and semantic (semantic relations) connections surrounding Taihu Lake and its primary connections, such as the Jiangnan Canal. Following this overview, Figure 5(c2) performs a granular deep-dive on this key sub-region, revealing the graph’s power to uncover deep knowledge chains. It traces how the Jiangnan Canal entity is semantically linked to profound historical concepts like Emperor Yang of Sui and World Heritage, while simultaneously retaining its validated spatial connections to Taihu Lake and Suzhou Creek. This validates the framework’s ability to move beyond simple co-occurrence to structure and reveal complex, multi-layered knowledge.

In summary, this multi-panel visualization (Fig 5) confirms that our LLM-Reflex-GeoKG framework successfully constructs a hybrid graph that is both structurally sound and functionally powerful. By transforming complex, multi-source geographic information data into this queryable knowledge network, it allows researchers, and the public to achieve a more intuitive and comprehensive understanding of the river network. Users can now intuitively grasp the foundational spatial hierarchy of the entire region (Fig 5a), understand the rich, validated semantic context of individual entities (Fig 5b), and perform multi-layered knowledge discovery on complex regional hubs (Fig 5c).

## Discussion

The primary objective of this study was to address key challenges in automatically constructing high-quality, domain-specific GeoKGs from multi-source data using LLMs, particularly concerning extraction accuracy, completeness, and the mitigation of knowledge hallucination. To this end, we proposed and validated a novel framework that integrates the Reflexion mechanism. Experimental results from a case study on the Yangtze River Delta region preliminarily confirm that by incorporating multi-stage processing and an iterative optimization strategy, our framework significantly improves extraction performance. Specifically, the method achieved net F1 score gains of 7.8% for Geographic NER over the single-stage baseline and 2.5% for Relation Extraction over the standard baseline. This quantitative evidence validates that the proposed framework provides a robust solution for leveraging LLMs to construct domain-specific knowledge graphs in settings that lack large-scale annotated data.

An in-depth analysis of our framework reveals that its effectiveness is underpinned by two core components. The Reflexion mechanism emerged as the primary driver of quality improvement, yielding significant performance gains in both zero-shot and few-shot settings, and proving particularly effective at enhancing factual accuracy. Concurrently, the multi-stage strategy employed during entity recognition proved instrumental in improving recall by decomposing the complex task into more manageable steps. Collectively, these findings validate our central design philosophy: that the performance of LLMs in complex, domain-specific extraction tasks can be systematically enhanced through a structured, iterative feedback loop. We also acknowledge the theoretical risk of error propagation inherent in the multi-stage strategy—specifically, that failing to identify an entity type in the initial scoping phase could preclude its subsequent extraction. However, our ablation analysis indicates that the benefits of ’attention focusing’ outweigh this risk, as evidenced by the 5.1% improvement in Recall compared to the single-stage baseline. Furthermore, the Reflexion mechanism effectively mitigates this propagation issue. Since the Evaluator reviews the extraction against the raw text, it can identify entities missed due to upstream filtering errors. The resulting feedback prompts the Executor to revisit the text with a corrected scope in subsequent iterations, thereby functioning as a dynamic error-correction layer.

In the context of existing research, our work addresses several persistent challenges through strategic differentiation. Unlike traditional supervised deep learning approaches that rely on extensive manual annotation and often struggle with heterogeneous data integration, our framework achieves competitive performance with minimal data examples (few-shot), significantly reducing deployment costs. Furthermore, in contrast to standard one-pass LLM extraction methods which are prone to factual hallucinations and lack precise geospatial grounding, our Reflexion-based approach actively suppresses errors through iterative self-correction without the need for computationally expensive parameter fine-tuning. Our method further distinguishes itself by explicitly integrating programmatically derived spatial relations from GIS analysis with LLM-extracted non-spatial relations. This hybrid approach aligns with and extends current research paradigms advocating for the integration of multi-source data to build spatially accurate and semantically rich GeoKGs.

Despite the promising results, this study has several limitations that warrant acknowledgment. First, the custom-annotated corpus used for quantitative evaluation is of a limited scale. Future work should validate the framework on larger and more diverse datasets to more comprehensively assess its generalization capabilities. Second, the Reflexion mechanism, while effective, introduces computational overhead. A key direction for future research will be to design more lightweight and efficient iterative feedback strategies without compromising efficacy. Third, regarding the entity alignment threshold (0.85), we acknowledge that this value was selected empirically to prioritize graph purity, lacking a comprehensive sensitivity analysis. While effective in the current study, this reliance on a fixed parameter represents a limitation. Future work will incorporate rigorous calibration experiments or adaptive thresholding mechanisms to better optimize the trade-off between precision and recall. Fourth, our work focuses exclusively on knowledge extraction from textual data; future extensions could incorporate knowledge fusion from multi-modal geographic data, such as remote sensing imagery and spatio-temporal trajectories. Looking ahead, the framework has significant potential for further optimization, such as applying it to other geographic regions or specific themes, and developing advanced downstream applications. These applications could include intelligent Question Answering (QA) systems and dynamic spatio-temporal reasoning that fully leverage the rich temporal information captured within our GeoKG, thereby unlocking its full value.

## Conclusion

This study addresses the core challenges of using LLMs for the automated construction of domain-specific Geographic Knowledge Graphs (GeoKGs): improving extraction accuracy, ensuring completeness, and mitigating knowledge hallucination. To tackle these issues, we propose LLM-Reflex-GeoKG, a Reflexion-augmented framework for GeoKG construction, and instantiate it on the Yangtze River Delta river network. Experimental results show that, compared with baseline methods, the proposed framework yields substantial improvements, achieving F1 scores of 0.898 for geographic entity recognition and 0.830 for relation extraction. Notably, this represents a significant performance gain of 7.8% in NER over the single-stage baseline of 0.820, and 2.5% in RE over the baseline of 0.805. These findings indicate that Reflexion-style self-reflection provides an effective path toward more reliable and controllable GeoKG construction in river-network scenarios.

Our analysis further reveals how the internal design of the framework contributes to these performance gains. The Reflexion mechanism, implemented through a collaborative dual-LLM architecture, systematically guides the iterative refinement of the model’s outputs. This process not only improves precision and recall under both zero-shot and few-shot conditions, but also effectively suppresses typical error modes, particularly ambiguous or inconsistent relation classification, driving a 2.5% improvement in the F1 score for relation extraction (from 0.805 to 0.830). In parallel, the multi-stage entity recognition strategy proves instrumental in boosting both accuracy and completeness. By effectively decomposing complex extraction tasks, it enhances the model’s ability to capture entities comprehensively while maintaining high precision through structured constraints, contributing to a 3.2% increase in NER F1 score compared to the single-stage baseline (0.820 to 0.852).

Overall, this study makes three main contributions. First, it proposes and validates LLM-Reflex-GeoKG, a Reflexion-augmented framework for automated GeoKG construction using LLMs. This framework offers a systematic methodology for building domain-specific GeoKGs, significantly reducing dependence on large-scale manual annotation. Second, it operationalizes the Reflexion mechanism in a geographic setting through generator–evaluator style interactions and multi-stage entity recognition, offering a practical blueprint for automating the construction of GeoKGs from complex geographic texts. Third, it delivers an empirically validated GeoKG for the Yangtze River Delta river network, demonstrating that the spatial structure of complex river systems can be seamlessly enriched with multi-dimensional semantic relations to diverse entities such as persons, events, and cultural entities, maintaining high fidelity and internal consistency.

In conclusion, these contributions offer a robust methodological framework for the broader field of GeoKGs. The proposed ontology, coupled with the Reflexion-based framework, can be easily adapted and referenced by the research community to construct GeoKGs in other specialized domains. Moreover, the flexible architecture of this framework paves the way for future community-driven extensions, such as the integration of multimodal data sources—specifically, high-resolution remote sensing imagery and vector topological data—thereby enhancing the comprehensiveness of spatial reasoning and enabling more advanced geospatial analysis.
